# Acute neuromuscular and cardiovascular effects of varying relative loads in cross-training modalities

**DOI:** 10.3389/fphys.2025.1636752

**Published:** 2025-08-21

**Authors:** Manuel Barba-Ruíz, Francisco Hermosilla-Perona, José Miguel Fernández-Asensio, Marzo Edir Da Silva-Grigoletto, Adrián Martín-Castellanos, Juan Ramón Heredia-Elvar

**Affiliations:** ^1^ Facultad de Ciencias Biomédicas y de la Salud, Universidad Alfonso X el Sabio (UAX), Madrid, Spain; ^2^ Facultad de Ciencias de la Vida y la Naturaleza, Universidad Nebrija, Madrid, Spain; ^3^ Department of Physical Education, Federal University of Sergipe, São Cristóvão, Brazil

**Keywords:** fatigue, pacing, performance, squat, strength

## Abstract

Training structures such as every minute on minute (EMOM), as many repetitions as possible (AMRAP), and rounds for time (RFT) have gained popularity for improving sport performance and general health. However, limited research exists on how different relative loads affect neuromuscular and cardiorespiratory responses. This study aimed to compare acute effects on heart rate (HR), mean propulsive velocity (MPV), velocity loss, and pacing in participants performing AMRAP, EMOM, and RFT using the same absolute but varying relative loads. Twenty-five participants with over a year of training experience in these structures performed squats, pull-ups, and shoulder press at varying relative intensities (<40%RM, 40–65%RM, and >65%RM). Results showed significant differences in MPV between modalities (p < 0.05), with RFT having higher MPV than AMRAP, especially at lower intensities (<40%RM). EMOM also had higher MPV than AMRAP, with minimal differences compared to RFT. Velocity loss patterns varied by intensity group, with AMRAP inducing higher intra- and inter-set losses than EMOM (p < 0.05). HR analysis revealed EMOM elicited the lowest HR values, followed by AMRAP and RFT, and a larger HR difference was noted in the lowest intensity group (p < 0.05). These findings suggest that prescribing relative loads, rather than absolute loads, is important for optimizing performance and managing fatigue in cross training.

## Introduction

In recent years, interest in high- and medium-intensity training methods has grown as a viable alternative to improving athletic performance and overall population health (Buchheit and Laursen, 2013; Feito et al., 2018a; Feito et al., 2018b). One of these training methodologies, initially affiliated with the CrossFit® brand, has given rise to a variety of practices collectively referred to as cross-training (Claudino et al., 2018; De-Oliveira et al., 2023; De-Oliveira et al., 2021). This term encompasses a wide range of activities, including weightlifting movements, gymnastics, and aerobic exercises, all designed to provide a higher level of cardiometabolic demands and a diverse range of movements (Maté-Muñoz et al., 2018). The appeal of these training modalities lies in their ability to challenge and improve various basic physical qualities such as strength, endurance, power, and flexibility, while facilitating physiological and metabolic adaptations (Buchheit and Laursen, 2013; Ferdinando, 2025; Rios et al., 2024).

Cross-modalities are characterized by their variable structure and emphasis on performing functional movements, often organized in exercise groups or “workouts of the day” (WOD) (Feito et al., 2018a). In terms of structure, these WODs generally adhere to one of two fundamental principles: a predetermined time duration or several specific tasks that must be executed within a period. In the first scenario, participants strive to complete as many repetitions or rounds as possible (AMRAP: as many rounds/reps as possible) within a specific time period, while in the second scenario (RFT: rounds for time), participants must finish a certain number of rounds in the shortest time possible. Lastly, the EMOM modality (every minute on the minute) involves performing a specific set of repetitions at 1-min intervals, with the remaining time used for rest before starting the next set (Barba-Ruíz et al., 2024; De-Oliveira et al., 2021). It is important to note that AMRAP and RFT formats may include self-selected rest periods, depending on the workout’s design and intensity demands.

Research into these training structures has been the focus of scientific inquiry to gain a deeper understanding of their acute and long-term physiological impacts on human health and performance (Jones et al., 2013). A specific area of interest has been the examination of cardiovascular response during cross-training modalities (Barba-Ruíz et al., 2024; Fernández et al., 2015; Maté-Muñoz et al., 2018; Tibana et al., 2018), traditionally assessed with the heart rate (HR) as an indicator of cardiovascular demand (Kang et al., 2022; Manresa-Rocamora et al., 2021). In addition to heart rate, considerable attention has been devoted to the vertical bar velocity as an index of exercise intensity and muscle fatigue (Sanchez-Medina and González-Badillo, 2011). The mean velocity during the concentric phase (MPV) of a strength movement has been shown as a useful variable to predict the relative intensity of each subject to different exercises or loads (González-Badillo and Sánchez-Medina, 2010). It represents the average velocity during the propulsive phase of a lift, where the acceleration of the barbell is positive, excluding the deceleration phase that occurs in certain exercises and low loads (González-Badillo and Sánchez-Medina, 2010). In addition, assessing propulsive velocity can measure relative intensity in these programs as the MPV achieved in relative load strongly correlates with the relative intensity (%RM); however, fatigue should be evaluated through velocity loss either within or between sets (Sanchez-Medina and González-Badillo, 2011). Several studies indicate strong correlations between mechanical and metabolic fatigue measurements, supporting the use of velocity loss to quantify peripheral fatigue during training (González-Badillo et al., 2017; Sanchez-Medina and González-Badillo, 2011). Therefore, measuring vertical bar velocity during cross-training sessions is an important aspect that must be considered to provide information about the workload and muscle fatigue faced by participants (Barba-Ruíz et al., 2024; De-Oliveira et al., 2021).

In these training structures, as in resistance exercises, muscular fatigue is a complex and multifactorial process, influenced by a variety of variables such as the nature of the exercises, the intensity of the training, and various physiological and psychological factors (Dominski et al., 2021). Within this context, intra- and inter-set velocity loss has been highlighted as a crucial indicator for evaluating neuromuscular fatigue (González-Badillo and Sánchez-Medina, 2010). This measure provides valuable information on how the neuromuscular system responds to the intense and varied loads imposed during cross sessions. Previous studies have demonstrated that velocity loss is closely related to neuromuscular fatigue, reflecting decreases in neural activation capacity and nerve impulse conduction velocity (Enoka and Duchateau, 2016; González-Badillo and Sánchez-Medina, 2010). This is particularly relevant in cross modalities, where athletes often perform fast movements that can lead to a rapid accumulation of fatigue. However, it is important to note that generalizing results may be limited due to the tendency of many studies to use small sample sizes and restricted experimental designs (Folland and Williams, 2007), underscoring the need for more comprehensive research in this field, tailored to the specific demands of cross training. Additionally, cross training has been characterized by using the same absolute intensity for all athletes regardless of their level (Escobar et al., 2017; Maté-Muñoz et al., 2018; Schlegel, 2020). This results in acute effects potentially differing among athletes as they handle loads with different relative intensities (Hottenrott et al., 2022). Similar to other sports characterized by high training loads (high volume or high loads), training organization should be based on relative intensity rather than absolute intensity.

Thus, the main objective of this research was to assess and compare the acute effects in HR, mean propulsive velocity, velocity loss, and pacing in participants with the same absolute loads but different relative intensities in cross modalities (AMRAP, EMOM, and RFT). It can be hypothesized that performing workouts using the same absolute load, but with differing relative intensities, will lead to distinct acute neuromuscular and cardiovascular responses depending on the training modality (AMRAP, EMOM, or RFT). In this context, individuals working at lower relative intensities are expected to exhibit a greater ability to regulate their pacing, resulting in more pronounced variations in movement velocity and fatigue between the different workout structures.

## Materials and methods

### Participants

A descriptive experimental cross-sectional study was conducted with a sample of 25 participants of both sexes (16 males and 9 females; age (years): 34.84 ± 6.56, weight (kg): 77.47 ± 12.43, height (cm): 174.43 ± 8.15), all of whom had at least 1 year of experience in cross training. All participants were athletes who trained regularly between three and five sessions per week and had experience competing at the regional level, and a few had participated in national-level events. To minimize variability, participants were instructed to avoid intense physical activity during the 24 h prior to each testing session and to abstain from caffeine consumption for at least 12 h before each trial. These participants did not report any injuries during the study period or in the 6 months preceding the start of the research. Data collection was carried out in accordance with the guidelines of the Declaration of Helsinki, to preserve human rights and to protect the privacy of the data subjects. The study was approved by the Alfonso X el Sabio Ethics Committee, and all subjects were informed of the inherent risks and benefits associated with study participation before signing informed consent forms.

### Procedure

The participants undertook three distinct cross routines using RFT, AMRAP, and EMOM across three separate days, with a full week of recovery between sessions. All assessments were conducted between approximately the same hours for each subject under similar environmental conditions (15 °C–20 °C and 30%–40% humidity).

Before each routine, participants completed a pre-routine test consisting of two squat repetitions with the prescribed absolute load detailed below. The mean MPV from these repetitions was recorded to determine the relative load that the absolute load represented for each subject. Additionally, a pause at maximal depth was incorporated to eliminate any stretch-shortening cycle effects (only in the pre-routine test).

Each routine consistently included three specific exercises: squats, pull-ups, and shoulder press. Importantly, the absolute loads and repetitions for these exercises remained the same for all subjects within each routine. However, males and females used different absolute loads: back squats (50 kg for males; 35 kg for females), butterfly pull-ups (body weight for both), and shoulder press (35 kg for males; 20 kg for females) using a power rack in the back squats and shoulder press. In addition, squat depth was determined based on the point at which the subject exhibited hip anteversion and loss of lumbar curvature during the descent (Straub and Powers, 2024). In these exercises, participants were instructed to perform repetitions as quickly as possible. Notably, pacing was autonomously regulated, driven by accumulated fatigue and the individual’s perceived exertion rather than being externally dictated.

The number of sets and rest periods varied based on the unique characteristics of each modality. During the AMRAP, participants were instructed to complete ten repetitions of each exercise within a 12-min timeframe. In the EMOM, each exercise was performed within a 1-min interval, including both work and rest periods. Participants completed 10 repetitions of each exercise within this timeframe, followed by rest until the next minute before proceeding to the next exercise. Lastly, in RFT, participants were tasked with completing half the number of rounds achieved in the AMRAP but in the shortest time possible.

The RFT session was always scheduled after AMRAP due to the design of the RFT protocol, which prescribed completing half the number of rounds achieved in the prior AMRAP session. The EMOM session was performed either before or after AMRAP, depending on participant availability. This structure was necessary to maintain consistency in workload prescription, while the 1-week separation between sessions ensured that the influence of the previous workouts on performance was negligible.

A heart rate monitor (Wahoo Tickr) was affixed to the chest using an elastic band for the collection of heart rate data (HR). These data were recorded every 30 s. Mean propulsive velocity (MPV) was recorded during the squat exercise during all the repetitions with a linear encoder (Vitruve encoder, Vitruve fit, Madrid, Spain).

### Data extraction

Participants were categorized into three relative intensity groups according to the mean propulsive velocity (MPV) achieved during the pre-routine test. They were categorized based on the MPV achieved and its correspondence with the %RM. (percentage of load corresponding to one-repetition maximum) (Sánchez-Medina et al., 2017): less than 40%RM (n = 9), 40–65%RM (n = 9), and greater than 65%RM (n = 7). These groups were created to compare participants for whom the same absolute loads represented different relative intensities.

MPV was recorded during the 10th repetition in all sets. Intra-set velocity loss was calculated as the difference between the 1st repetition (or the fastest repetition) and the 10th repetition (or the slowest repetition) within each set. Inter-set velocity loss was determined by comparing the 1st repetition of each set across rounds (e.g., the 1st rep of set 1 vs. the 1st rep of set 2). All velocity loss values were expressed as percentages. For pacing purposes, the mean velocity across all repetitions was also calculated.

### Statistical analysis

The mean ± standard deviation (SD) was obtained for descriptive analysis of the study variables. A *post hoc* power analysis (G*Power 3.1) indicated that with a sample size of 25 participants and three comparison groups, f = 0.65 was approximately 80%. The normality of the data distribution was assessed and confirmed through the Shapiro–Wilk test.

A two-way repeated measures ANOVA was performed to analyze the differences in MPV between each of the subjects and the routine (subject×routine), splitting the results into each relative intensity group (<40% RM, 40–65%RM, >65%RM). In addition, a two-way ANOVA was conducted to assess the differences between each relative intensity group between modalities (intensity group×routine). A Bonferroni *post hoc* analysis was conducted to assess the differences in each factor in all the analyses carried out. Effect size as partial eta squared (η_p_^2^) values was employed to present the magnitude of differences with 0.01, 0.06, and above 0.15 thresholds for small, medium, and large effect sizes, respectively (Cohen, 1988).

All analyses were conducted using SPSS v.29.0 statistical software for Mac Os (IBM SPSS Statistics) and GraphPad Prism 10 software. The significance level was set at p < 0.05.

## Results

**FIGURE 1:**
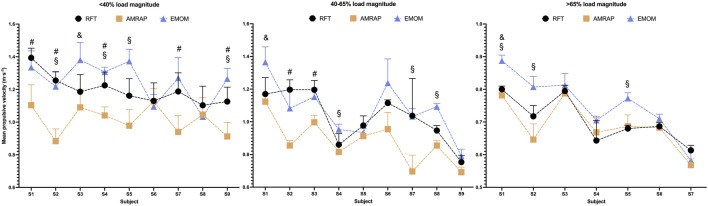
Differences in MPV in each subject according to the modality and load magnitude groups. Note: #: differences between AMRAP and RTF; &: Differences between RFT and EMOM; §: differences between EMOM and AMRAP. All the differences showed were statistical significant for α>0.05.

Mean MPV comparisons between modalities in each subject are reported in Figure 1. Two-way ANOVA results present statistical differences and a large effect size (p < 0.001; η_p_2 = 0.484). The *post hoc* results showed that the <40%RM and 45–65%RM groups, the MPV values present more statistical differences between AMRAP and the other modalities (lower MPV in AMRAP) than do the results of the >65%RM group. Specifically, the modalities with large differences between them are the RFT and AMRAP, which show higher mean MPV values for RFT (p < 0.05). This seems to be a tendency in which these differences are more pronounced (<40%RM) in the groups with lower load magnitude. In addition, the EMOM presents a higher mean MPV than AMRAP (p < 0.05); however, these differences tend to be present in the same way in all load magnitude groups. Finally, lower differences were found between EMOM and RFT. Interestingly, the mean MPV values are higher in EMOM for the >65%RM group, but this tendency is not observed in the other groups.

**FIGURE 2:**
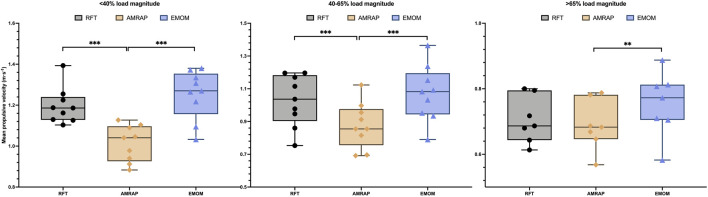
Mean propulsive velocity between modalities in each load magnitude group. Note: *p < 0.05; **p < 0.01; ***p < 0.001.

The comparisons between the mean MPVs in each load magnitude group display the same tendency between modalities (Figure 2). Two-way ANOVA results present statistical differences and a medium effect size (p < 0.001; η_p_2 = 0.063). MPV values between RFT and EMOM show no significant differences between them in any load magnitude groups. However, in the AMRAP modality, the subjects present lower MPV values with significant differences with the other modalities (p < 0.01), except for EMOM in the >65%RM load magnitude group.

**FIGURE 3:**
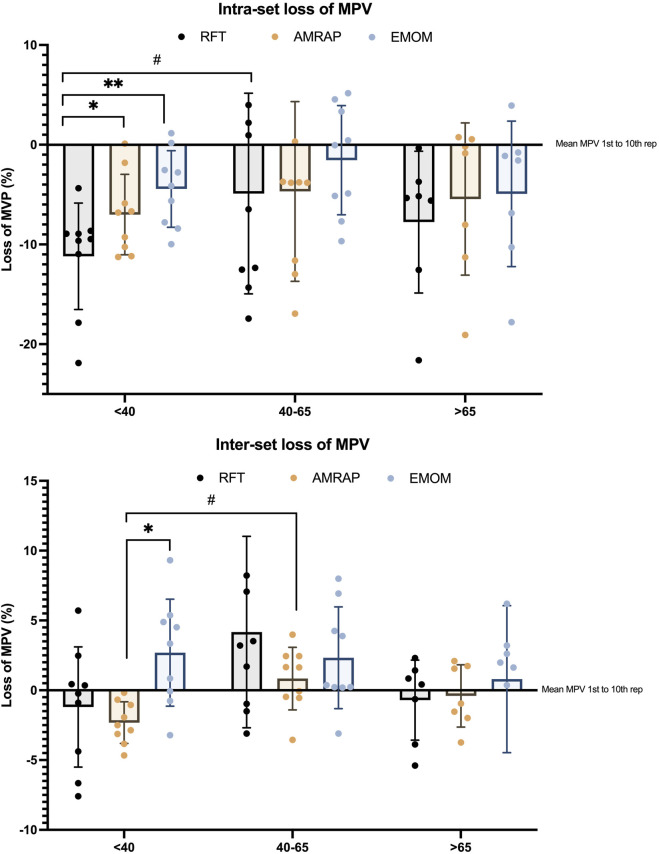
Differences in loss of MPV between groups and modalities. Note: * indicates differences between modalities in each group: *p < 0.05; **p < 0.01; ***p < 0.001; #p < 0.05; # indicates differences between the same modalities across groups #p < 0.05; ##p < 0.01; ###p < 0.001.

The fatigue analysis and the intra- and inter-set loss of velocity are displayed in Figure 3. Two-way ANOVA results present statistical differences and a medium effect size (p < 0.001; η_p_^2^ = 0.12). *Post hoc* analysis of intra-group differences showed only differences in the <40%RM group. RFT showed higher intra-set velocity losses than AMRAP and EMOM with statistically significant differences (p < 0.05 and p < 0.01, respectively). Inter-set velocity loss was lower in EMOM (showing positive values, higher velocities in the subsequent sets) than AMRAP, which showed higher MPV loss values (p < 0.05). A comparison between the same modality across the groups showed that the intra-set MPV loss in RFT was higher in the <40%RM group than in the 40–65%RM group (p < 0.05). In addition, the inter-set analysis reports differences between the <40%RM and 40–65%RM groups in the AMRAP modality.

**FIGURE 4:**
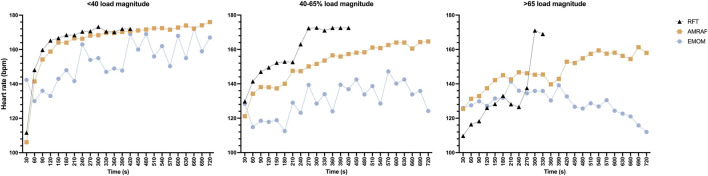
Descriptive heart rate data according to modality and load magnitude groups.

**FIGURE 5:**
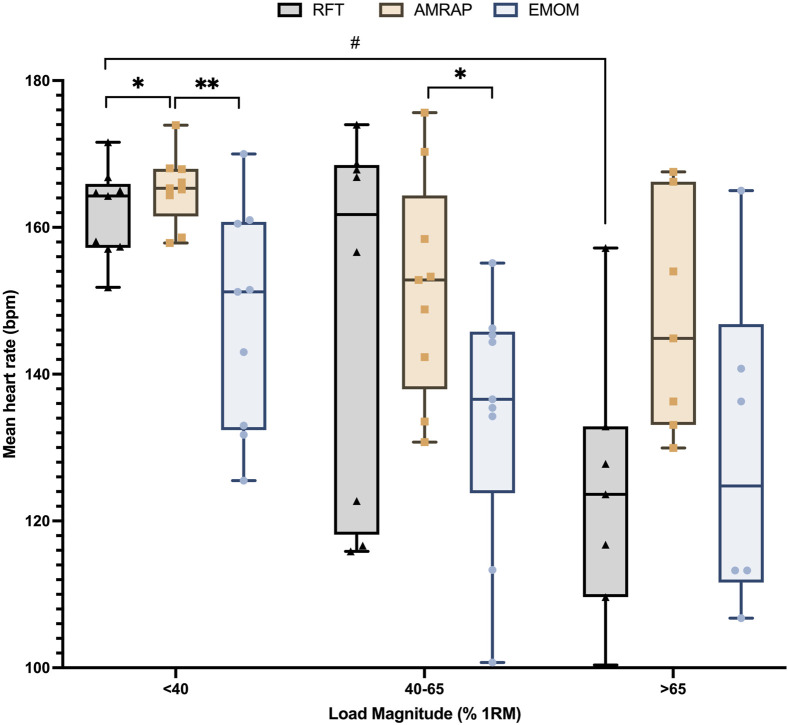
Comparative heart rate results according to modality and load magnitude groups. Note: * indicates differences between modalities in each group: *p < 0.05; **p < 0.01; ***p < 0.001; #p < 0.05; # indicates differences between the same modalities across groups #p < 0.05; ##p < 0.01; ###p < 0.001.

Heart rate analysis is shown in Figure 4, which indicates that EMOM was the modality in which the subjects reported lower HR values in all groups, followed by AMRAP and finally RFT. A comparison between groups and modality found some interesting differences (Figure 5). The AMRAP modality presented higher HR than RFT (p < 0.05) and EMOM (p < 0.01) in the <40%RM group; however, this finding was not observed in the other groups. The EMOM modality presented the lowest HR values and also reported significant differences with AMRAP in the 40–65%RM group (p < 0.05).

**FIGURE 6:**
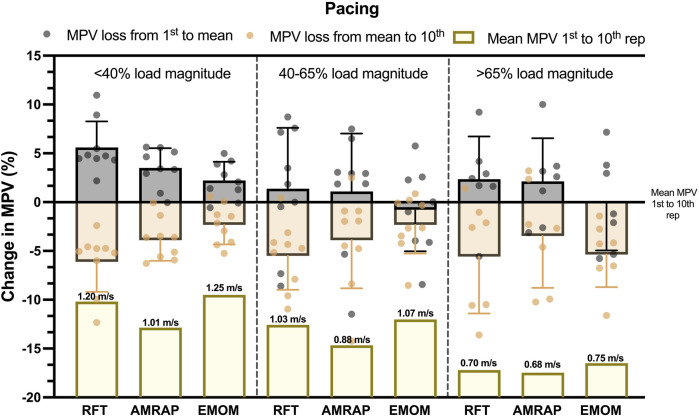
Descriptive pacing distribution in execution velocity in each load magnitude group and modality. Note: MVP loss from 1st to mean: MPV loss between the first repetition and the mean velocity of the 10 repetitions; MPV loss from mean to 10th: loss of MPV between the mean velocity of the 10 repetitions and the 10th repetition; Mean MPV 1st to 10th rep: mean velocity across all the repetitions in each set.

Finally, the effort distribution in each modality was analyzed with the pacing that the subject adopted in each modality (Figure 6). First, in the <40%RM group, it can be observed that RFT showed higher variations between the 1st MPV and the 10th MPV. In this group, subjects performed the repetitions with a wider range of velocity (less regulation in the effort). However, AMRAP showed smaller lower variations and a lower mean MPV, indicating better pacing regulation by the athletes. Interestingly, EMOM presented the lowest MPV variation, probably due to a lower fatigue achieved. In the remaining groups, a lower pacing profile can be observed, in which the athletes have fewer possibilities to manage their execution velocities and, therefore, lower MPV variation. Nevertheless, the same tendency with a higher variation in RFT following by AMRAP and EMOM can be observed in the 40–65%RM and <65%RM groups.

## Discussion

The main objective of this research was to assess and compare the acute effects on heart rate, mean propulsive velocity, velocity loss, and pacing in participants with the same absolute and different relative loads in cross modalities (AMRAP, EMOM, and RFT).

The findings of this study suggest that varying relative loads, even when the absolute load remains the same, can offer different stimuli to athletes depending on the routine. Additionally, training volume was structured with a fixed 10 repetitions per set for each exercise, while the number of rounds varied by modality: 4 rounds in EMOM, 4–5 rounds in RFT, and 8–10 rounds in AMRAP. Although no specific volume analysis was conducted, the total repetitions were comparable between EMOM and RFT, whereas AMRAP allowed for higher variability and overall volume depending on individual pacing. This fact was previously reported by other research, which indicated that athletes with large-volume modalities manage the velocity voluntarily and apply less force in each repetition to reduce accumulated fatigue throughout the routine (Barba-Ruíz et al., 2024; De-Oliveira et al., 2021). Another aspect that needs to be considered is that under different high-intensity training regimens over a short period of time (4–5 min), athletes can achieve the same neuromuscular performance as EMOM configurations with the same volume but with periods of rest included in the routine and larger blocks of time (12 min). Interestingly, these behaviors were the same in athletes with different relative loads.

The reported velocity losses suggest that, first, inter-set velocity loss is generally unaffected by relative intensity and training modalities, except in the lower relative intensity group, where AMRAP shows significantly larger velocity losses than EMOM. Additionally, lower relative intensity groups in AMRAP experience greater velocity losses than groups where the absolute load represents higher relative intensities. This could be due to the fact that athletes with lower relative intensities present higher MPVs and can manage the velocities across the sets in a more extended way, especially in longer duration routines. Second, intra-set velocity losses follow a similar pattern, with differences between modalities only observed in the lower relative intensity group. These distinct patterns of velocity loss between AMRAP and EMOM at lower intensities are likely related to differences in pacing strategies inherent to each modality (Barba-Ruíz et al., 2024).

Several studies have identified different patterns in pacing strategies during these modalities. In this sense, in higher volume modalities, athletes can self-regulate the force applied and adopt pacing strategies across different modalities (Barba-Ruíz et al., 2024; De-Oliveira et al., 2021; Mangine and Seay, 2022). In athletes for whom the absolute loads represent lower relative intensity, RFT exhibited the highest variations in MPV between the 1st and 10th repetitions, suggesting a less regulated effort and a more pronounced range of velocities. This aligns with previous research indicating that in time-constrained tasks, athletes tend to adopt an “all-out” pacing strategy (De-Oliveira et al., 2021). AMRAP, on the other hand, demonstrated smaller variations and a lower mean MPV, indicating higher pacing regulation by athletes. This observation is consistent with studies showing that in tasks with a fixed duration, athletes often employ a more conservative, evenly paced approach (Mangine and Seay, 2022). Finally, EMOM presented the smallest variations in MPV, possibly due to lower accumulated fatigue, which may be attributed to the structured rest periods inherent in this modality (De-Oliveira et al., 2021). As relative intensity increased, a general trend toward lower pacing profiles emerged, with athletes having fewer opportunities to manage the velocity. This finding supports the concept of intensity-dependent pacing, where the ability to self-regulate diminishes as the relative intensity increases. In this context, higher pacing profiles are more achievable when absolute loads are moderate to low and for athletes with greater strength levels. However, if the relative intensity of each exercise is very high, the ability to self-regulate velocity throughout the repetitions becomes limited and constrained (González-Badillo et al., 2017; Sanchez-Medina and González-Badillo, 2011).

Finally, HR response across different modalities and relative intensity groups reveals different behaviors. EMOM consistently elicited the lowest HR values across all intensity groups, followed by AMRAP, with RFT generally inducing the highest HR responses. This hierarchy aligns with previous research, which shows that different cross workout structures can significantly impact physiological responses (Feito et al., 2018b). The lower HR values observed in EMOM may be attributed to the short and regular rest intervals that allow for partial recovery (Tibana et al., 2018). This structured rest may also influence other metabolic factors, such as the recovery of phosphocreatine stores and the clearance of fatigue-inducing metabolites like lactate, which can help sustain high force production and delay the onset of fatigue (Escobar et al., 2017; Fernández et al., 2015). Conversely, the absence of rest in AMRAP protocols promotes continuous muscle activation, leading to the accumulation of fatigue, related metabolites, and a progressive increase in lactate concentrations (Escobar et al., 2017).

Interestingly, the comparative analysis between relative intensity groups and modalities revealed that AMRAP elicited higher HR responses than both RFT and EMOM in the athletes with lower relative loads. Nevertheless, the differences were smaller in the moderate and higher relative load groups, suggesting that as relative load increases, the cardiovascular response becomes similar across different modalities. This finding highlights the fact that, at higher relative intensities, the specific modality may become less influential in determining cardiovascular output, as the load itself becomes the primary factor influencing the exercise response.

A limitation of this study is the sample size, which was determined by the maximum number of participants who met the inclusion criteria. While this allowed for the collection of meaningful data within a practical field, a larger sample could strengthen the statistical robustness of the findings and improve their overall representativeness. Also, due to the real-world, ecological nature of the study, it was not feasible to monitor or control participants’ broader nutritional habits or hydration status. Finally, the absence of physiological markers such as blood lactate, cortisol, or creatine kinase limited our ability to explore metabolic and neuromuscular fatigue in greater depth.

## Conclusion

This study demonstrates that athletes with lower relative loads exhibited greater differences in key variables, including HR, MPV, and velocity loss, across different cross modalities. Athletes with lower relative loads demonstrated a greater possibility to adjust and control their MPV, resulting in more variations in pacing and performance across routines. Conversely, athletes with higher relative loads exhibited more consistent responses across modalities, as the increased load limited their capacity to self-regulate velocity and adjust pacing. In this context, the differences between modalities seem to be largely influenced by relative load, with higher loads reducing the neuromuscular and cardiovascular effects observed in athletes with lower relative loads across different training modalities. Based on the findings, it is recommended that coaches and athletes prescribe cross-training modalities using relative rather than absolute loads to optimize performance and better manage fatigue. Additionally, a training structure should be selected according to the athlete’s strength profile. EMOM sessions may be more appropriate for individuals with lower relative strength, as the built-in rest intervals help reduce velocity loss and fatigue. In contrast, AMRAP formats may induce greater fatigue and are therefore better suited for stronger or more experienced athletes.

## Data Availability

The raw data supporting the conclusions of this article will be made available by the authors, without undue reservation.
